# Methodological considerations in longitudinal morphometry of traumatic brain injury

**DOI:** 10.3389/fnhum.2013.00052

**Published:** 2013-02-26

**Authors:** Junghoon Kim, Brian Avants, John Whyte, James C. Gee

**Affiliations:** ^1^Moss Rehabilitation Research InstituteElkins Park, PA, USA; ^2^Department of Radiology, University of PennsylvaniaPhiladelphia, PA, USA

**Keywords:** longitudinal, power, bias, magnetic resonance imaging, sparse canonical correlation analysis

## Abstract

Traumatic brain injury (TBI) has recently been reconceptualized as a chronic, evolving disease process. This new view necessitates quantitative assessment of post-injury changes in brain structure that may allow more accurate monitoring and prediction of recovery. In particular, TBI is known to trigger neurodegenerative processes and therefore quantifying progression of diffuse atrophy over time is currently of utmost interest. However, there are various methodological issues inherent to longitudinal morphometry in TBI. In this paper, we first overview several of these methodological challenges: lesion evolution, neurosurgical procedures, power, bias, and non-linearity. We then introduce a sensitive, reliable, and unbiased longitudinal multivariate analysis protocol that combines dimensionality reduction and region of interest approaches. This analysis pipeline is demonstrated using a small dataset consisting of four chronic TBI survivors.

## Introduction

Traumatic brain injury (TBI) triggers a cascade of events that lead to long-term neuropathological and behavioral consequences. Recently, more and more researchers have been conceptualizing TBI as a chronic disease with dynamic and evolving recovery and degeneration processes (e.g., Masel and Dewitt, 2010). Among the post-injury changes in moderate to severe TBI, widespread volume reduction in brain parenchyma, frequently called diffuse atrophy, is probably most prominent and significant (Bigler, 2005; Povlishock and Katz, 2005). An important mechanism of this diffuse change is Wallerian degeneration due to traumatic axonal injury, which may continue months and years after injury. Describing the spatial and temporal characteristics of these degenerative morphological changes may provide important clues for the mechanisms underlying individual differences in functional recovery or decline, ultimately contributing to development of better treatment. In mild TBI, due to limited sensitivity of conventional cross-sectional approach, objective evidence of brain injury has been difficult to obtain. Employing a within-subject longitudinal design, future morphometry studies in this group may reveal evidence of sutle brain changes over time. Ideally, the neural degeneration processes can be tracked longitudinally by conducting repeated assessments using sensitive and reliable *in vivo* non-invasive imaging methods such as structural magnetic resonance imaging (MRI). However, only a handful of studies with a limited number of assessment time points are available to date (e.g., Ross, 2011). Because an increasing number of longitudinal studies are expected in the future, it would be useful to review the methodological challenges inherent in analyzing longitudinal imaging data at the early stage of this area of research. The first goal of this paper is to briefly overview the following methodological considerations in longitudinal morphometry of TBI: lesion evolution (structural changes in lesion over time), neurosurgical procedures (surgery on central nervous system), power (probability to detect changes when they are truly present), bias (directional error in parameter estimation), and non-linearity (relationship that cannot be described by the first degree equations). Another purpose of this article is to introduce a novel method that combines dimensionality reduction and region of interest approaches (Avants et al., 2010a), which intends to serve as an example of sensitive, reliable, and unbiased longitudinal multivariate change detection protocols. This analysis pipeline will be demonstrated and validated using a pilot TBI dataset. We focus on structural MRI measures to quantify neurodegeneration such as volume change and cortical thickness indices. However, the principles and conclusions from this paper may be generalized to data using other imaging modalities and techniques such as diffusion tensor imaging and functional MRI. In addition, the same logic can be applied to quantify neural regeneration.

## Methodological challenges in longitudinal morphometry of TBI

### Lesion evolution

From the moment of injury, the brain goes through numerous structural changes, both focal and diffuse in nature (Gennarelli and Graham, 2005; Povlishock and Katz, 2005; Kubal, 2012). We here review two types of lesion characteristics that can confound progressive atrophy measurement in a longitudinal imaging study of TBI.

#### Edema

Posttraumatic edema is accumulation of water in the intracellular and/or extracellular spaces of the brain. The precise time course for human posttraumatic cerebral edema has yet to be determined. If this “brain swelling” is diffuse, it effectively leads to an overestimation of brain volume. Therefore, if the first assessment in a longitudinal study is in the acute phase, researchers must exercise great caution because the changes due to the initial stabilization of the brain are confounded with subsequent atrophy. This results in overestimation of the amount of atrophy over time. If one's primary interest is in the post-acute phase, the first measurement can be done after edema is completely resorbed, e.g., 1–2 months after the injury (e.g., Bendlin et al., 2008; Ng et al., 2008; Sidaros et al., 2009).

#### Focal encephalomalacia

If the brain suffers from various types of bleeding (hematoma and hemorrhage) or bruises (contusions), local tissue abnormalities appear in the imaging. The size of the lesion in the acute phase is known to evolve (increase or decrease) over time depending on various factors (e.g., Chang et al., 2006). In the post-acute phase, if the cells surviving the acute phase develop atrophy, the size of focal encephalomalacia is likely to grow slowly. Changes in focal lesions over time make it difficult to accurately quantify diffuse atrophy, especially in the perilesional areas. One way to deal with focal abnormalities is to exclude participants with focal injuries or conduct a subgroup analysis by dividing participants into two groups according to the presence of focal lesions. Typically, a lesion volume threshold is employed because it is practically infeasible to exclude the brains with focal lesions of any size. Using different thresholds for cortical and subcortical lesions may make sense considering that smaller lesions in the subcortical regions often have more detrimental functional consequences. However, excluding those with focal lesions will introduce a severity bias. Another way to control the effects of focal lesion is to restrict the analysis only to the areas where no focal lesions are found for all participants. To achieve this, lesion masks are built (typically manually) for each brain and then combined to form a “lesion frequency map” (e.g., Levine et al., 2008; Kim et al., 2010). Recent efforts to develop automatic lesion detection algorithms have shown some success in reducing the burden of manual lesion drawing (e.g., Hillary and Biswal, 2009; Ghosh et al., 2011).

### Neurosurgical procedures

Various neurosurgical procedures, performed to stabilize the injured brain (e.g., evacuation of hematomas/hemorrhages, controlling intra-cranial pressure, etc.), are invasive and cause temporary and/or permanent alterations of the brain. Edema and glial scarring from surgical procedures typically need weeks to months to stabilize, making it challenging to separate the unique effect of surgery and true neural degeneration on longitudinal imaging measures. If neurosurgery leaves focal alterations, they may be dealt with similar methods used for focal encephalomalacia (see section “Focal encephalomalacia”). However, research is lacking in this area. Most neurosurgeries are done during the acute phase. However, procedures such as cranioplasty can be done in the post-acute phase and require careful follow-up. For example, it was recently reported that a significant portion of patients who undergo cranioplasty develop fluid collection underneath the site of operation (Chang et al., 2010; Lee et al., 2011), which may distort brain volume measurement. Even more important is the issue of late cranioplasty and how having skull replaced part-way into a longitudinal study might affect the results.

### Power

Statistical power in structural neuroimaging depends on various factors including effect size, measurement error, method of multiple comparison correction, and sample size. Unfortunately, in longitudinal studies of patients with moderate to severe TBI, sample size is typically small due to participant attrition and numerous exclusion criteria including metal implants, lesion characteristics, and patient movement in the MRI scanner. In mild TBI, while fewer participants' data are lost, the effect size of longitudinal change is expected to be relatively small. Therefore, adopting a sensitive and reliable imaging analysis protocol becomes more crucial to achieve powerful longitudinal change detection. There are various ways to quantify longitudinal changes in the imaging data (e.g., Bosc et al., 2003; Holland et al., 2012). They can be classified into two broad categories: (1) regions-of-interest (ROI) or segmentation based approaches and (2) deformation or registration based methods. Here we briefly discuss these two change detection methods in the context of power.

One way to improve detection power in small imaging datasets is to use a limited number of a priori ROIs and measure longitudinal changes within those ROIs. Typically, ROIs are constructed at each time point for each individual by an “expert” human rater or an automatic segmentation algorithm. The majority of previous longitudinal imaging studies in TBI used this approach (e.g., Ng et al., 2008; Warner et al., 2010; Xu et al., 2010). Assuming that the size and location of the ROIs are on target, this approach is likely to increase statistical power. However, reliable a priori hypotheses are frequently unavailable due to the lack of existing research and thus potentially important changes in unselected regions can be missed. In addition, due to heterogeneous injury mechanisms and lesion characteristics in TBI, using the same set of a priori ROIs across different samples may not be ideal. An important limitation of manually defining ROI is that human raters do not reproduce the same results when measurement is repeated, introducing repeat measurement error. Maintaining high intra- and inter-rater reliability costs a substantial amount of time and effort. Automating ROI construction using computer algorithms is a potential solution. However, automatic segmentation of brain structures, especially when a lesion is present, still remains one of the most challenging tasks.

To overcome some of the limitations of the ROI or segmentation based approach, some researchers have adopted deformation based methods of change detection. These methods do not rely on the researcher to identify specific ROIs and are well-suited to exploratory studies. In deformation or tensor based morphometry (DBM/TBM; e.g., Ashburner et al., 1998; Ashburner and Friston, 2000), one image (e.g., a patient's) is directly warped to the other (e.g., a template) using non-linear deformable spatial registration and the resulting deformation fields are used for quantification of volume differences between the two images. Reliability of this approach is, in general, much higher than manual drawing. Deformation based methods may be used for serial studies as well. To quantify longitudinal changes, a subject's brain at one time point may be directly warped to the same person's brain at later time point (e.g., Sidaros et al., 2009). Directly comparing images effectively bypasses the repeat measurement error issue, offering advantages in terms of statistical power. For TBI, large deformation registration schemes (for an introduction, see Ashburner, 2007) should be used to allow detection of a wide range of volume changes (Kim et al., 2008). Custom or population-specific templates can also help increase detection power (Lepore et al., 2007). Many large deformation algorithms, some of them using diffeomorphisms, are freely available and their performance was recently compared in a large-scale evaluation study (Klein et al., 2009).

More recently, multivariate pattern analysis (MVPA) has been employed to increase detection power over univariate approaches, particularly in fMRI studies (e.g., Haxby et al., 2001). In the last section, we will illustrate an image analysis pipeline that uses MVPA to interrogate longitudinal TBI effects.

### Bias

Bias, a directional error in parameter estimation, can arise in longitudinal morphometry when data from different time points are not treated equivalently. In that sense, avoiding bias in the ROI/segmentation based approach is relatively straightforward because the same system of measurement can be applied to the data at each time point. For example, one can keep the expert raters blinded to the order of measurements or even randomize the images from different time points. In DBM/TBM, however, due to the fact that the images from two or more time points are directly compared, there is a possibility that the images undergo systematically different processing steps.

The issue of bias in longitudinal DBM/TBM has recently become the focus of discussion among researchers investigating the trajectory of atrophy using the Alzheimer's disease Neuroimaging Initiative database (ADNI; www.loni.ucla.edu/ADNI). Partly due to this discussion, several nice summaries on this topic are available in the literature (Holland et al., 2012; Hua et al., 2011; Thompson and Holland, 2011) and they enumerate sources of bias and rules to follow to prevent those biases from affecting the estimate of longitudinal changes. One important issue is designating images at each time point as the source (i.e., image to be warped) or the target (i.e., image to be warped to). Symmetric or inverse-consistent registration methods should be used to avoid bias from asymmetric registration processes. An inverse-consistent or symmetric registration is an algorithm that yields the same correspondences between the images from two time points when the order of them is switched (e.g., Avants et al., 2008). If inverse-consistency cannot be guaranteed by the registration algorithm itself, symmetry may still be achieved by first measuring the changes in both directions independently and then averaging them (Thompson and Holland, 2011). In addition, all images from different time points should undergo the same number of interpolations. For example, Yushkevich and colleagues recently showed that distributing interpolation equally across all of a subject's images is critical for eliminating bias (Yushkevich et al., 2010).

### Non-linearity

To our knowledge, most existing longitudinal morphometry studies in TBI have had only two measurement points. As studies with multiple time points emerge, it would be important to consider the potential non-linear nature of the post-injury atrophy. First of all, true biological non-linearity needs to be distinguished from the spurious non-linearity caused by bias. A good example is a controversial study conducted by Hua and colleagues (2010), in which structural atrophy of AD patients was quantified between the baseline and four follow-up scans using TBM. Cumulative atrophy was determined by warping all of the follow-up images to the same baseline image. As a result, the authors found an unexpected non-linearity—i.e., a rapid jump of the atrophy rate between the baseline and the first follow-up point, with more linear increase thereafter. This jump was due to the fact that this study design confounded both interpolation effects and atrophy effects, which have a similar magnitude (approximately 1% change). Subsequent re-analysis studies showed that this trend was replicated even in healthy controls and that a large portion of the bias could be corrected by using inverse-consistent registration methods (Hua et al., 2011; Thompson and Holland, 2011) which interpolate every time point in the same way.

Allowing for the possibility of true biological non-linearity in the atrophy rate after TBI, the next question becomes the exact shape of non-linear degeneration and how much individual variability exists. Growth curve analysis, which is known to be capable of accommodating many mathematical functions, missing data points, variable time intervals, and individual differences, may be a fruitful approach to explore. Mixed effects models have similar advantages.

## Example of longitudinal multivariate morphometry: a pilot study

### Rationale

MVPA has gained acceptance for its ability to combine the benefits of both prior-constrained ROI studies and the exploratory nature of DBM/TBM. This multivariate approach reduces the multiple comparisons problems by clustering regions together in an automated way (known as dimensionality reduction in machine learning) and then allowing hypotheses to be tested on this reduced set of areas. We here hypothesize that, even in a small, heterogeneous TBI sample, there are common areas across individuals that undergo neurodegeneration and that can be detected as regions of correlated white matter (WM) and gray matter (GM) changes (Avants et al., 2010b).

### Method

**Figure 1:**
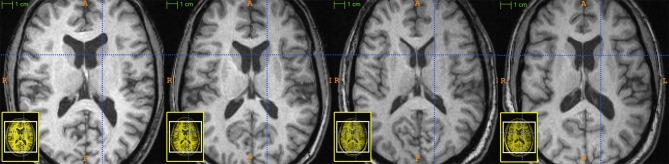
**Four TBI survivors' representative axial scans at the baseline**.

In this study, we used best-practice pre-processing to determine two quantitative measures (i.e., cortical thickness and WM volume change) throughout the whole brain in four survivors of diffuse TBI (average age at baseline = 38.3) serial T1 data (Figure 1). The average time post-injury at baseline was 6.6 months and the average assessment interval was 15.8 months. We then applied a dimensionality reduction technique, sparse canonical correlation analysis for neuroimaging (SCCAN), to obtain a limited number of ROIs that are sensitive and specific to longitudinal change that is related across tissues (Avants et al., 2010a,b). As reviewed above, quantitative longitudinal analyses must be conducted with unbiased techniques if the image-based measurements are to be interpreted physically (Yushkevich et al., 2010). Thus, we used Advanced Normalization Tools (ANTs) and a population-specific template (Avants et al., 2010c; Reuter et al., 2012) to compute the unbiased deformable mapping between each subject's baseline and follow-up image. The resulting diffeomorphic mapping quantifies longitudinal volume changes. We also employed prior-based spatiotemporal maximum a posteriori image segmentation to extract change in the cortical GM over time (Avants et al., 2011) and to identify cortical thickness alterations in each subject. This processing protocol leads to two complementary measures that may be used to assess atrophy—i.e., WM volume change via Jacobian determinant and GM cortical thickness. Thickness is, in general, more sensitive than volumetric measures because it incorporates tissue-specific information (Hutton et al., 2009). Both measures were annualized.

We hypothesized that, in chronic TBI, spatially distinct but covarying atrophy would be observed in both WM and GW and that this atrophy rate would be greater than zero. In summary, the procedure was as follows: (1) Apply the unbiased longitudinal mapping methods (Avants et al., 2010a; Yushkevich et al., 2010); (2) Quantify the annualized volumetric change in WM and the annualized atrophy in GM; (3) Employ SCCAN to identify four localized regions of GM and four correlated and localized regions in WM to be tested for significant atrophy; (4) Use the one-sample *t*-test with false discovery rate correction (FDR) to determine if the atrophy in SCCAN-identified regions is significant. We arbitrarily limited the number of regions to 4 for WM and 4 for GM considering the small number of participants.

### Results and discussion

**Figure 2:**
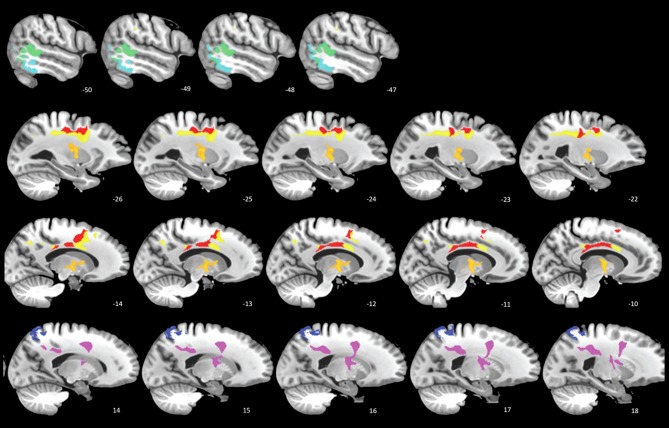
**SCCAN reveals multiple cortical and white matter regions of longitudinal atrophy.** Cortical areas (cool colors) include posterior temporal lobes, posterior cingulate, and superior parietal lobe. The white matter and deep gray matter (warm colors) regions includes the thalamus (orange, second row), primary motor tract, and the mid- and posterior bodies of the corpus callosum.

Seven of eight regions (4 WM, 3 GM) were significant at the FDR-corrected *p* < 0.1 level (Figure 2). In line with previous TBM studies with larger samples (Kim et al., 2008; Sidaros et al., 2009), these regions included thalamus, corpus callosum, and posterior cingulate (see Figure 2 legend for the complete list). In the most significant region (*p* < 0.015), the estimated atrophy rates for WM and GM were 7.3 ± 3.9% and 4.2 ± 1.8%, respectively (mean ± SD of the amount of atrophy in percentage). FDR-corrected *p* < 0.05 is also achievable if only the most highly correlated regions are tested.

This preliminary study reveals significant longitudinal atrophy patterns after correction for multiple comparisons despite the very few subjects involved. Three main points emerge from this design: (1) significant and localized regions with atrophy may be identified by employing MVPA in longitudinal morphometry of TBI; (2) unbiased approaches are essential for identifying quantitative atrophy measures and retain enough power to be effective in small cohorts; (3) this paradigm is easily extended to include additional modalities and will be more reliable with additional subjects. In addition, future research comparing the current method with alternative approaches are warranted.

### Conflict of interest statement

The authors declare that the research was conducted in the absence of any commercial or financial relationships that could be construed as a potential conflict of interest.
